# A Severe Case of Neuralgia

**Published:** 1891-02

**Authors:** A. V. Syontagh

**Affiliations:** Budapest


### A SEVERE CASE OF NEURALGIA.

Dr. A. V. Syontagh, Budapest.

(*Allg. hom. Zeit.*, No. 17, 1890.)

Madame M., fifty years old, the daughter of an old-school physician, well nourished, of quiet, phlegmatic temperament, chlorotic as a girl, as the wife always healthy and especially free of any nervous troubles even during menstruation, sterile. In her forty-sixth year, without known cause, she was attacked with a severe neuralgia in irregular paroxysms, mostly beginning during the night and continuing many hours, attacking at first the head, then the abdomen and finally the womb. These attacks repeated themselves daily for eighteen months. Many high authorities of the old school were consulted, and every known treatment of the old school had its chance, but everything failed. She had already lost twenty-seven kilogrammes, and her mind became weak. In mere despair Homœopathy was thought of, and Dr. Hausmann (the author of *Ursachen und Bedingungen der Krankheit*) called in, and a few weeks sufficed to restore her health, which she enjoyed till 1886, when she again suffered from the same neuralgia, caused by catching cold during a heavy winter storm. At five in the morning, hardly a quarter of an hour sooner or later, the severe headache woke her up, continued in all its force during the forenoon, and gradually ceased in the afternoon. The attending physicians could benumb the pain with their hypodermics of Morphine, but could not cure the case. Hausmann had died during the interval and thus Syontagh was finally called in about February, who found the woman tortured by the most severe pains, starting in the neck and occiput, spreading over the vertex to the forehead and radiating into the eyes. The continuous pains, from the surface inwardly, are boring, pressing, off and on radiating and tearing, never hammering; more on the right than on left side, and by motion and especially by the touch of the scalp; in fact, touch anywhere is very painful, especially in back; abdomen, thighs are hyperæsthetic, hands and feet never. Face pale during the attack, eyes neither red nor weeping, nor photophobic; pupils normal; temporal artery normally pulsating; pulse small, hard, moderately frequent; increased salivation. Nux-vomica was selected as most similar to the symptoms, 3x with some Strychnine$^{3x}$ and the hypodermics strictly forbidden. Next morning the attack began at seven A. M., two hours later, and ceased at noon. Salivation increased. Next day the attack lasted only four hours and was milder. On the 24th no headache, only some precordial oppression, more profuse salivation. On the 27th and 28th she had only very mild attacks and then they ceased, but she still complained of this hyperæsthesia and salivation, and it seemed as though the neuralgia had only changed places, for many a morning she complained of pains in the chest and abdomen or in the bladder and womb. Symptoms were: Sensation of constriction in the throat, oppression in chest, precordial anguish, constricting gastralgia, hiccough, intestinal colic, frequent and painful desire to micturate and passing large quantities of nervous urine; constant desire to defecate, with sensation of a ball in rectum; spasmodic pains in the womb without leucorrhœa and great sensitiveness of left ovarian region to pressure. Salivation steadily the same; gynæcological exploration and examination of urine with negative results. All other functions normal. Syontagh compared Mercur., Belladonna, Cocculus. Conium, Natrum-muriaticum, Nux-vomica, Secale, Sepia, Veratrum, and finally settled on Nux-vomica, which gave some relief, Belladonna and Conium nearly removed everything, except the ptyalism, which yielded in a few days to Pilocarpinum-mur. 6th dilution, and henceforth she seemed to be able to enjoy uninterrupted health.

Why Syontagh did not think of Cedron is astonishing. No other remedy has such clock-like regularity in its paroxysm and in the treatment of neuralgic affections. Cedron takes in my practice a high rank, and it has especially this clock-like periodicity or prosopalgia and nervous headache, also profuse ptyalism, constriction in throat, hiccough, spasms in stomach and bowels, profuse urination and all troubles worse after coitus; a state sometimes found in hysterically-inclined females. We may have either profuse perspiration or salivation. Cedron would have come nearer to be a simillimum than Nux-vomica or Belladonna. When will our physicians gain confidence enough to use the higher dilutions, for in nervous disorders they are certainly more advisable than the low ones, which are again better when the vegetative system suffers; Nux$^{2c}$ or M and tincture of time would have acted more promptly. Another remedy which loomed up before my mind's eye in such a chronic migraine was Silicea, and which would have followed well to eradicate this psoric condition. S. L.

